# Correction: Recent developments in hydrogels containing copper and palladium for the catalytic reduction/degradation of organic pollutants

**DOI:** 10.1039/d5ra90028j

**Published:** 2025-03-19

**Authors:** Jaber Dadashi, Mohammad Ali Ghasemzadeh, Masoud Salavati-Niasari

**Affiliations:** a Catalysts and Organic Synthesis Research Laboratory, Department of Chemistry, Iran University of Science and Technology Tehran Iran; b Department of Chemistry, Qom Branch, Islamic Azad University Qom Iran ma.ghasemzadeh@iau.ac.ir qasemzade.a@gmail.com; c Institute of Nano Science and Nano Technology, University of Kashan Kashan Iran

## Abstract

Correction for ‘Recent developments in hydrogels containing copper and palladium for the catalytic reduction/degradation of organic pollutants’ by Jaber Dadashi *et al.*, *RSC Adv.*, 2022, **12**, 23481–23502, https://doi.org/10.1039/d2ra03418b.

The authors regret an error in Scheme 14 and in the caption for Fig. 6.

The incorrect image was inserted previously, and Scheme 14 should have been:

**Scheme 1:**
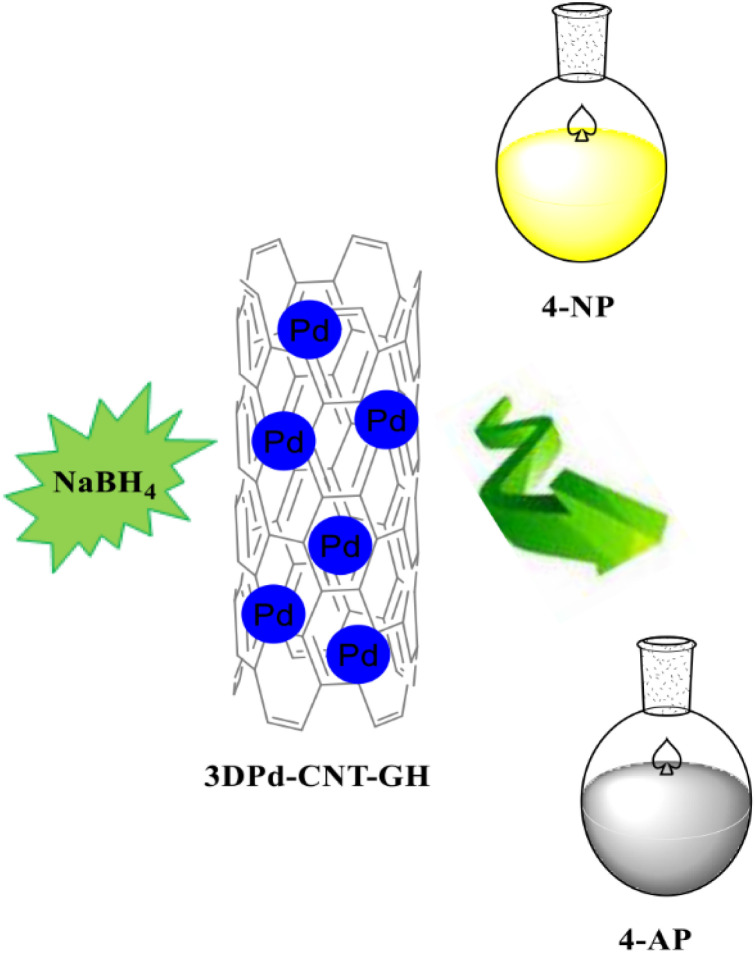
Reduction of 4-NP in the presence of 3D Pd–CNT–GH.

In addition, the caption for Fig. 6 should have cited ref. 118. The caption for Fig. 6 should have read:

**Fig. 6** Preparation and catalytic application of PEI–Ag/PEI–Pd composites and PEI hydrogel. This <svg xmlns="http://www.w3.org/2000/svg" version="1.0" width="10.166667pt" height="16.000000pt" viewBox="0 0 10.166667 16.000000" preserveAspectRatio="xMidYMid meet"><metadata> Created by potrace 1.16, written by Peter Selinger 2001-2019 </metadata><g transform="translate(1.000000,15.000000) scale(0.014583,-0.014583)" fill="currentColor" stroke="none"><path d="M240 840 l0 -40 -80 0 -80 0 0 -40 0 -40 40 0 40 0 0 -80 0 -80 -40 0 -40 0 0 -40 0 -40 80 0 80 0 0 40 0 40 40 0 40 0 0 -40 0 -40 80 0 80 0 0 40 0 40 -40 0 -40 0 0 80 0 80 40 0 40 0 0 40 0 40 -80 0 -80 0 0 40 0 40 -40 0 -40 0 0 -40z"/></g></svg> gure has been reproduced from ref. 118 with permission from the ACS. CC-BY license.

The Royal Society of Chemistry apologises for these errors and any consequent inconvenience to authors and readers.

